# TTK activates Akt and promotes proliferation and migration of hepatocellular carcinoma cells

**DOI:** 10.18632/oncotarget.5295

**Published:** 2015-09-19

**Authors:** Xing Liu, Weijia Liao, Qing Yuan, Ying Ou, Jian Huang

**Affiliations:** ^1^ National Engineering Center for Biochip at Shanghai, Shanghai, China; ^2^ Shanghai-MOST Key Laboratory for Disease and Health Genomics, Chinese National Human Genome Center at Shanghai, Shanghai, China; ^3^ Key Laboratory of Systems Biomedicine (Ministry of Education) and Collaborative Innovation Center of Systems Biomedicine, Shanghai Center for Systems Biomedicine, Shanghai Jiao Tong University, Shanghai, China; ^4^ Hepatology Institute of Guilin Medical University, Guilin, Guangxi Zhuang Autonomous Region, China

**Keywords:** hepatocarcinogenesis, TTK gene, cell migration, demethylation, Akt

## Abstract

Hepatocellular carcinoma (HCC) is one of the most malignant cancers with poor clinical outcome. The protein kinase human monopolar spindle 1 (hMps1/TTK) gene expression is significantly increased in HCCs. However, its contributions to hepatocarcinogenesis remain unclear. In this study, we found that TTK was overexpressed in 77.63% (118/152) HCC specimens. Elevated TTK expression positively correlated with large tumor size and presence of the portal vein tumor thrombus (PVTT). Demethylation in its promoter increased TTK expression in HCC. *In vitro* assays revealed that TTK not only promoted cell proliferation and anchorage-independent growth, but also cell migration. Subsequent investigations revealed that TTK activated Akt/mTOR pathway in a p53 dependent manner. We also found that TTK specific kinase inhibitor AZ3146 could decrease HCC cell growth. In conclusion, TTK contributes to HCC tumorigenesis via promoting cell proliferation and migration. It may serve as a novel biomarker and a potential target in HCC cancer therapy.

## INTRODUCTION

Hepatocellular carcinoma (HCC) is one of the most malignant tumor worldwide, causing more than 746,000 deaths per year (http://globocan.iarc.fr/Pages/fact_sheets_cancer.aspx). Conventional clinical and pathological indexes are used in adjudgement of HCC stages and outcomes but limited with low accuracy. The molecular mechanism of HCC carcinogenesis still remains unclear [1]. Thus, it is important to seek novel biomarkers and molecular targets of HCC.

Protein kinases comprise enzymes with catalytic activity and transduces functional signalings as key regulators of critical cellular processes [2]. Dysregulation of kinases in tumors contribute to carcinogenesis and is associated with tumor progression and clinical outcome [3]. Inhibitors are screened targeting on kinases for molecular cancer therapy [4]. In HCC, sorafenib is demonstrated to be the only effective small inhibitor for patients at advanced stage [5]. It still remains a critical challenge to identify relevant molecular targets and effective small inhibitors in liver cancer therapy.

The human monopolar spindle 1 (hMps1/TTK) gene (NM_003318) locates on chromosome 6q13-q21 and encodes a dual serine / threonine and tyrosine protein kinase. TTK is essential for the mitotic checkpoint and improper chromosome attachments [6, 7]. Elevated TTK level leads to amplified centrosomes, hyperactivated SAC and chromosome instability, thus contributing to tumorigenesis [8]. Potential diagnostic value of TTK in certain carcinomas including undifferentiated thyroid carcinoma, triple-negative breast cancer and lung cancer was observed [9–11]. Specific inhibitors of TTK were screened using high throughput *in vitro* kinase assays, yielding AZ3146, MPI-0479605 and Mps1-IN-1 in inhibiting proliferation of cervical carcinoma cell Hela and colon carcinoma cell HCT-116, respectively [12–14]. In HCC, TTK is overexpressed and correlated with age, HBsAg, Edmondson tumour grade and satellite lesion [15, 16]. However, its role and molecular mechanism still need more investigation.

In this study, we first investigated the clinical significance and prognostic value of TTK in HCC. Exploration of the methylation status of TTK promoter revealed the potential mechanism of its up-regulation in HCC specimens. We then examined its effects on cell proliferation and migration. In addition, we attempted to inhibit HCC cell growth using TTK specific inhibitor. Finally, we explored the signaling pathways activated by TTK in HCC. Our study highlights the significance and underlying mechanism of TTK in HCC carcinogenesis.

## RESULTS

### TTK mRNA expression is frequently increased in HCC

**Figure 1:**
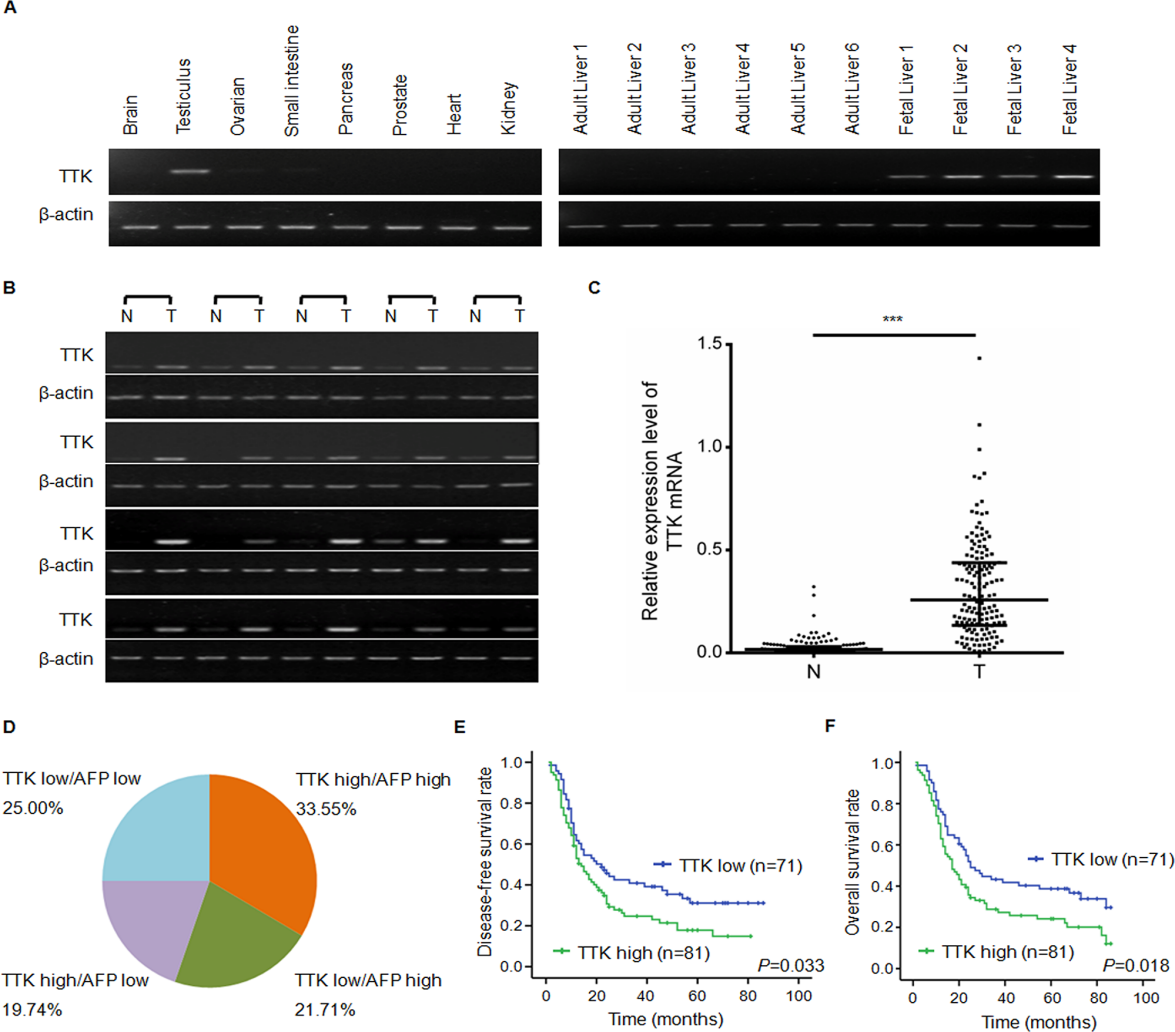
TTK expression patterns and clinical significance of TTK mRNA level in HCC specimens **A.** Results of the semi-quantitative RT-PCR analyses of TTK in 8 normal tissues, 6 normal liver tissues and 4 fetal liver tissues. **B.** Representitive results of TTK mRNA level in 20 pairs of HCC specimens (T) and their adjacent non-tumorous liver tissues (N) **C.** TTK mRNA level in 152 paired HCC (T) and adjacent non-tumorous liver tissues (N) measured by QuantiGene Plex assay (***, *P* < 0.001). The lines represented 75%, medium and 25% of TTK mRNA value, from the top down, respectively. **D.** The pie chart showed the percentages of the 4 groups divided on the basis of TTK and AFP expression levels in 152 HCC specimens. TTK high: TTK mRNA level is greater than or equal to 0.226 in HCC specimens; TTK low: TTK mRNA level is less than 0.226 in HCC specimens; AFP high: AFP protein level is greater than or equal to 200 μg/l in peripheral blood; AFP low: AFP protein level is less than 200 μg/l in peripheral blood. **E.** Disease-free survival rates of HCC patients with different TTK expression levels. **F.** Overall survival rates of HCC patients with different TTK expression levels.

We first assessed the TTK expression profile in several human tissues by RT-PCR and demonstrated that TTK was predominantly expressed in the testis, with very low levels in the ovarian and the small intestine. Notably, TTK was highly expressed in fetal liver while absent in normal human liver (Figure 1A). In 20 cases of HCC tumor specimens, TTK mRNA expressions were dramatically increased, comparison to their paired adjacent non-tumor specimens (Figure 1B). To confirm this result, TTK mRNA level was examined in all 152 patients with HCC by QuantiGene Plex 2.0^®^ assay. Compared with the corresponding non-cancerous livers, 118 of the 152 HCC cases (77.63%) showed an over 5-folds increase of the TTK mRNA level (*P* < 0.001, Figure 1C).

We then assessed whether the TTK mRNA level was associated with clinical annotations. As shown in Table 1, high expression of TTK was significantly correlated with high level of serum AFP (*P* = 0.050), large tumor size (*P* = 0.006), advanced TNM-stage (*P* = 0.020), more presence of portal vein tumor thrombus (PVTT, *P* = 0.003) and distant metastasis (*P* = 0.004), which indicates the important role of TTK in HCC tumor growth and metastasis.

**Table 1:** Correlation between the clinicopathologic variables and TTK expression in HCC

Clinical character	variable	No. of patients	mRNA		χ^2^	*P* value
			low *n* (%)	high^a^ *n* (%)		
Age (years)	<50	75	25 (33.3)	50 (66.7)	10.644	**0.001**
	≥50	77	46 (59.7)	31 (40.3)		
Gender	Male	136	60 (44.1)	76 (55.9)	0.490	0.070
	Female	16	11 (68.8)	5 (31.2)		
Family history	No	129	58 (45.0)	71 (55.0)	1.048	0.367
	Yes	23	13 (56.5)	10 (43.5)		
HbsAg^b^	Negative	32	15 (46.9)	17 (53.1)	0.000	1.000
	Positive	120	56 (46.7)	64 (53.3)		
AFP^c^ (μg/l)	<200	68	38 (55.9)	30 (44.1)	4.158	**0.050**
	≥200	84	33 (39.3)	51 (60.1)		
Median size (cm)	<3	28	14 (77.8)	4 (22.2)	7.917	**0.006**
	≥3	134	57 (42.5)	77 (57.5)		
Cirrhosis	No	19	5 (26.3)	14 (73.7)	3.628	0.084
	Yes	133	66 (49.6)	67 (50.4)		
Tumor number	Single	101	44 (43.6)	57 (56.4)	1.197	0.304
	Multiple	51	27 (52.9)	24 (47.1)		
Wine-drinking	No	63	24 (38.1)	39 (61.9)	3.208	0.099
	Yes	89	47 (52.8)	32 (47.2)		
Smoking	No	67	32 (47.8)	35 (52.5)	0.909	0.635
	Yes	85	39 (45.9)	46 (54.1)		
TNM stage^d^	I–II	61	36 (59.0)	25 (41.0)	6.199	**0.020**
	III–IV	91	35 (38.5)	56 (61.5)		
PVTT	No	111	60 (54.1)	51 (45.9)	8.915	**0.003**
	Yes	41	11 (26.8)	30 (73.2)		
Distant metastasis	No	136	69 (50.7)	67 (49.3)	8.408	**0.004**
	Yes	16	2 (12.5)	14 (87.5)		
Recurrence	No	111	51 (45.9)	60 (54.1)	0.097	0.855
	Yes	41	20 (48.8)	21 (51.2)		

a high of TTK mRNA expression in HCC specimens was designed as greater than or equal to 0.226

b HBsAg, hepatitis B surface antigen

c AFP, alpha-fetoprotein

d TNM, tumor-node-metastasis

We compared the TTK mRNA level in HCC specimens with the serum AFP protein level and found they were not totally consistant. TTK was significantly overexpressed in 30 cases of HCC specimens (30/152, 19.74%) with negative serum AFP protein level (<200 μg/l). Considering the original proportion of cases (84/152, 55.26%) with positive serum AFP level (≥200 μg/l), the combined use of TTK with AFP would improve the sensitivity of diagnosis up to 75% (Figure 1D). Interestingly, TTK expression was notably increased in persons under age 50 (Table 1, *P* = 0.001). In the age segmentation of under 50, the combined use of TTK and serum AFP would improve the diagnostic rate from 61.33% to 85.33% (supplymentary Table 1). These data suggest that TTK might be a novel biomarker of HCC especially in patients under age 50.

We then assessed the prognostic value of TTK by analyzing the disease-free survival rate and the overall survival rate. We found that high TTK expression was significantly associated in Kaplan-Meier analysis model with poor disease-free survival rate (*P* = 0.033, Figure 1E) and overall survival rate (*P* = 0.018, Figure 1F). Taken together, these results imply that TTK might be a potential marker in diagnosis and prognosis.

### Demethylation of TTK promoter contributes to its overexpression in HCC

**Figure 2:**
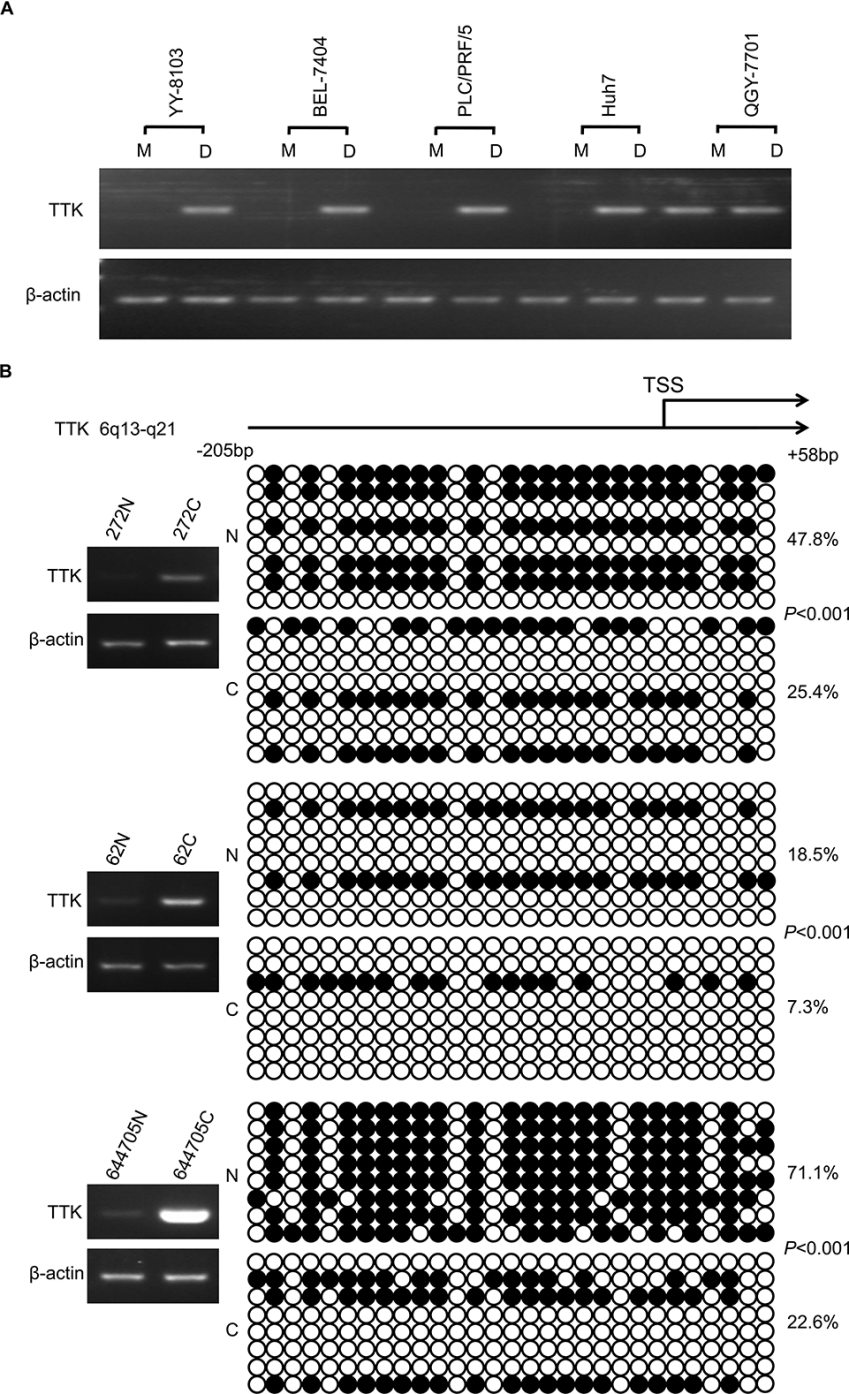
The DNA methylation status of TTK promoters in HCC **A.** YY-8103, BEL-7404, PLC/PRF/5, Huh7 and QGY-7701 cells were treated with DAC (work concentration: 2.0 μM). The expression of TTK was evaluated by RT-PCR. M: untreated cells used as control. D: DAC treated cells. **B.** Results of bisulfite sequencing in 3 cases of HCC specimens with obviously elevated TTK mRNA level. Each circle indicates a CpG dinucleotide within the CpG island in the promoter. Black: methylated; white: unmethylated.

TTK mRNA expression is reported to be up-regulated due to copy number variation (CNV) in triple-negative breast cancer [11]. However, considering the frequent deletion of chromosome 6q in HCC [17], we speculates whether epigenetic modifications contribute to the dysregulation of TTK in HCC. Several types of HCC cell lines were treated with demethylating agent 5-aza-2′-deoxycytidine (decitabine, DAC) at a working concentration of 2 μM for 72 h. Compared with the no drug treated control, TTK mRNA level increased dramatically in YY-8103, BEL-7404, PLC/PRF/5 and Huh7 cell lines, while it was not observed in only QGY-7701 (Figure 2A).

To confirm the demethylation status of TTK promoter, bisulfite sequencing was conducted in 3 pairs of HCC tumor and non tumor specimens. An elevated TTK expression was observed in these 3 cases of HCC tumor specimens. The sequencing data showed that in all 3 cases of HCC specimens, the amount of the 5-methylcytosine (5-mC) was significantly decreased than in their paired non-cancerous liver tissues (*P* < 0.001, Figure 2B).

### TTK stimulates HCC cell proliferation, colony formation and migration *in vitro*

**Figure 3:**
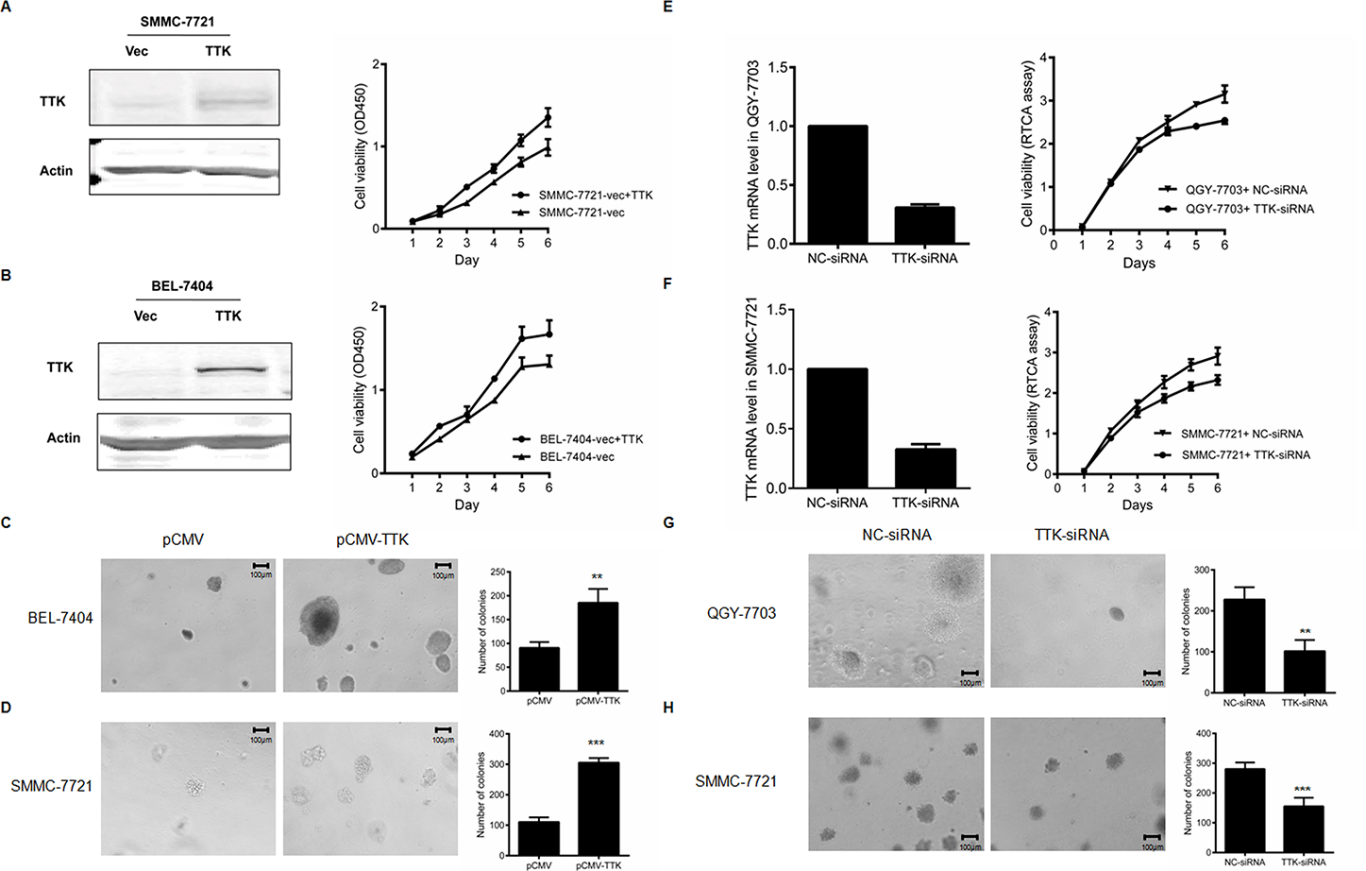
Effects of TTK on HCC cell growth **A.** and **B.** The representative proliferation curve of SMMC-7721 (A) and BEL-7404 (B) after the ectopic expression of TTK. Points, mean (*n* = 6); bars, SD. **C.** and **D.** The colony formation in soft agar after the ectopic expression of TTK in BEL-7404 (C) and SMMC-7721 (D) cells. The numbers of colonies in the histograms represented mean values in triplicate (mean ± SD) (**, *P* < 0.01; ***, *P* < 0.001). **E.** and **F.** The proliferation curves of QGY-7703 (E) and SMMC-7721 (F) after the knockdown of TTK by specific siRNA. Cell proliferation curves were monitered by the RTCA assay. The points represented mean value of 2 times of independent experiments; bars, SD. **G.** and **H.** The colony formation in soft agar after the in QGY-7703 (G) and SMMC-7721 (H) cells.

To evaluate the function of TTK on tumorigenesis in HCC, we then observed the effects of TTK on cell proliferation and anchorage-independent cell growth. Ectopic TTK expression vectors were transfected into BEL-7404 and SMMC-7721 cells. The exogenous expression was validated by western blotting. Cells grew significantly faster than those transfected with the empty control vectors (Figure 3A and 3B). Given that anchorage-independent growth is one of the key hallmarks of cell transformation and malignancy [18], colony formation in soft agar assay was performed. The results demonstrated that exogenous TTK expression stimulated anchorage-independent cell growth (*P* < 0.05, Figure 3C and 3D).

To confirm the effects of TTK on HCC cell growth, knockdown experiments were then performed in QGY-7703 and SMMC-7721 cells. The knockdown efficiency of the designed siRNA was measured by quantitative real-time PCR, with NC-siRNA served as a control. Our data showed that decreased TTK expression inhibited both anchorage-dependent (Figure 3E and 3F) and anchorage-independent HCC cell proliferation (*P* < 0.05, Figure 3G and 3H).

**Figure 4:**
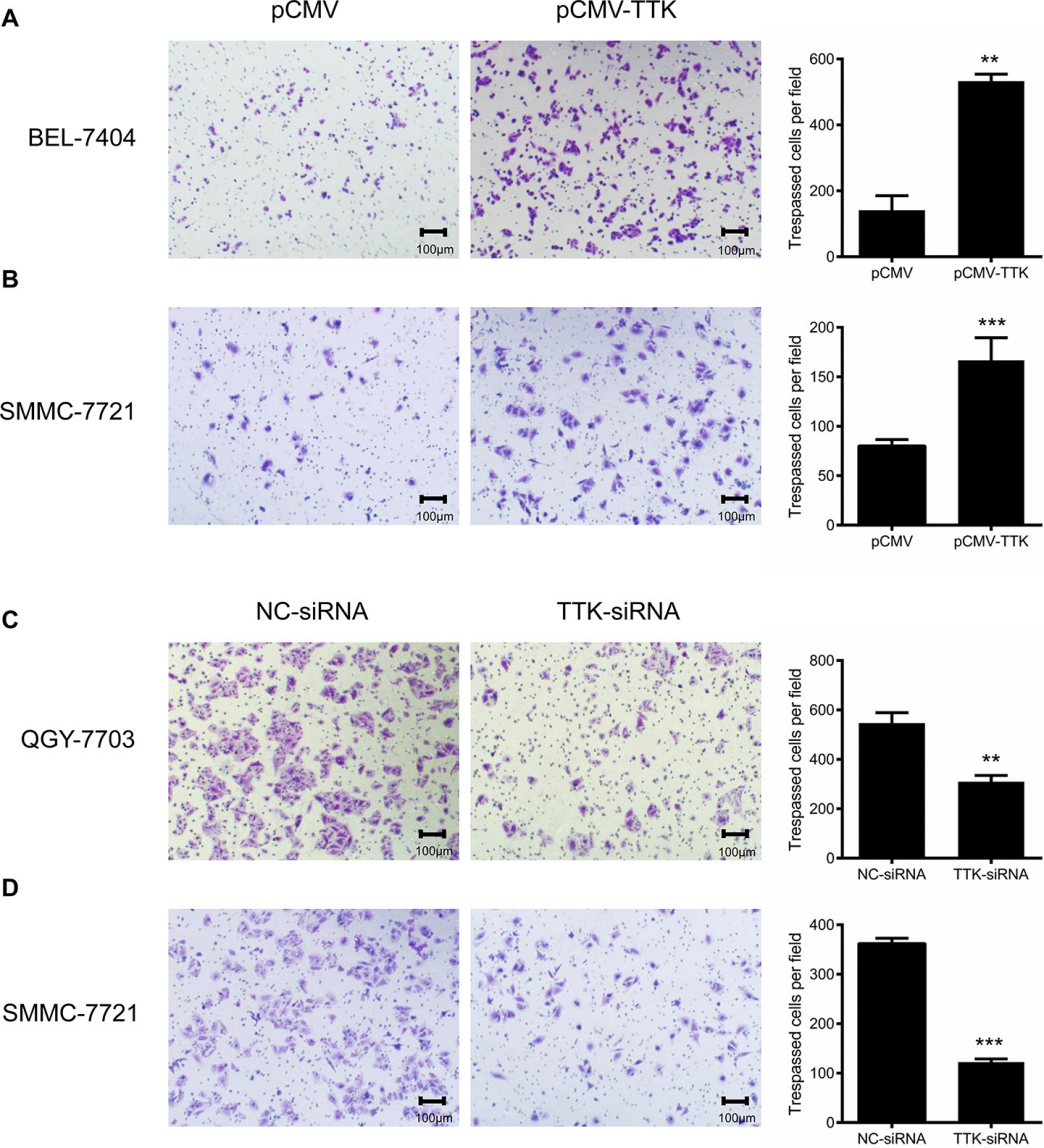
Effects of TTK on HCC cell migration Representative images of HCC cell migration in transwell assay. **A.** and **B.** Exogenous expression of TTK increased BEL-7404 (A) and SMMC-7721 (B) cell migration. **C.** and **D.** Reduction of endogenous TTK expression with specific siRNA decreased QGY-7703 (C) and SMMC-7721 (D) cell migration. The number of tespassed cells in the histograms represented mean values per field (from 3 fields, mean ± SD) (**, *P* < 0.01; ***, *P* < 0.001).

We further evaluated whether TTK induced HCC cell migration with transwell assay. BEL-7404 and SMMC-7721 cells with an ectopic TTK expression migrated faster than controls (*P* < 0.05, Figure 4A and 4B). On the contrary, transfection of TTK siRNA in QGY-7703 and SMMC-7721 significantly decreased cell migration (*P* < 0.05, Figure 4C and 4D). All results suggest that TTK contributes to tumorigenesis through promoting cell growth, colony formation and migration of the existing HCC cells.

### TTK activates Akt mediated pathways in HCC cells

**Figure 5:**
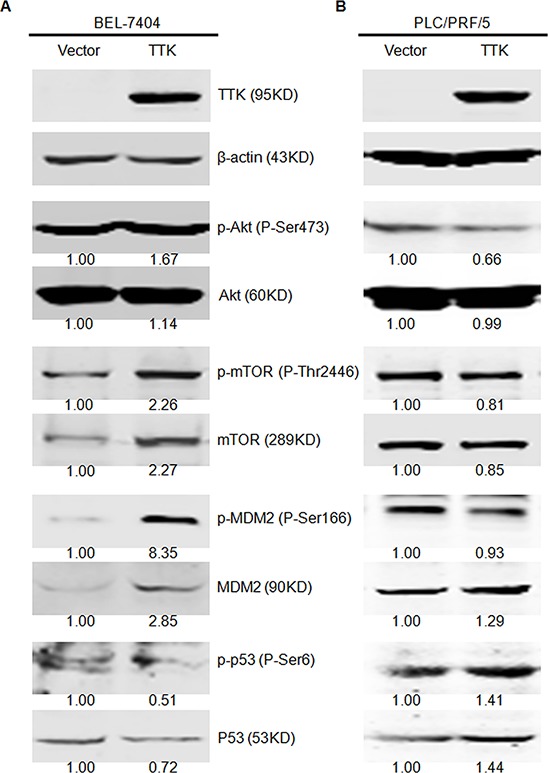
TTK overexpression induces the phosphorylation of Akt relative signaling pathways **A.** Overexpression of TTK enhanced the phosphorylation of Akt (P-Ser473), mTOR (P-Thr2446), and MDM2 (P-Ser166) and inhibited the phosphorylation of TP53 (P-Ser6) in SMMC7721 cell line. **B.** Overexpression of TTK did not enhance the phosphorylation of Akt (P-Ser473), mTOR (P-Thr2446) and MDM2 (P-Ser166) and promoted the phosphorylation of TP53 (P-Ser6) in PLC/PRF/5 cell line. Quantification of each band was normalized to β-actin and indicated by the numbers above the corresponding panels.

To investigate the underlying mechanism of TTK in stimulating HCC cell proliferation, we first estimated its effects on activating Akt and mTOR kinases, the signaling pathways contributing to cell growth and anchorage-independent colony formation. Using western blotting, we found that phosphorylation levels of Akt (P-Ser473) and mTOR (P-Thr2446) kinases were significantly increased in BEL-7404 cells with TTK exogenous expression (Figure 5A). We then assessed the phosphorylation levels of Akt downstream protein kinases MDM2 (P-Ser166) and p53 (P-Ser6), key regulators of the cell apoptosis signaling pathway. The phosphorylation of MDM2 was markedly enhanced, whereas the phosphorylation of p53 was significantly suppressed in TTK transfected BEL-7404 cells (Figure 5A). Interestingly, similar activations were not observed in p53 mutant PLC/PRF/5 cells (Figure 5B). It indicates that Akt/mTOR signaling responds differently due to the status of p53. These results demonstrate that TTK stimulates the malignance of HCC cells via Akt/mTOR and MDM2/p53 activation in a p53 dependent mechanism.

### TTK kinase inhibitor AZ3146 impares HCC cell viability

**Figure 6:**
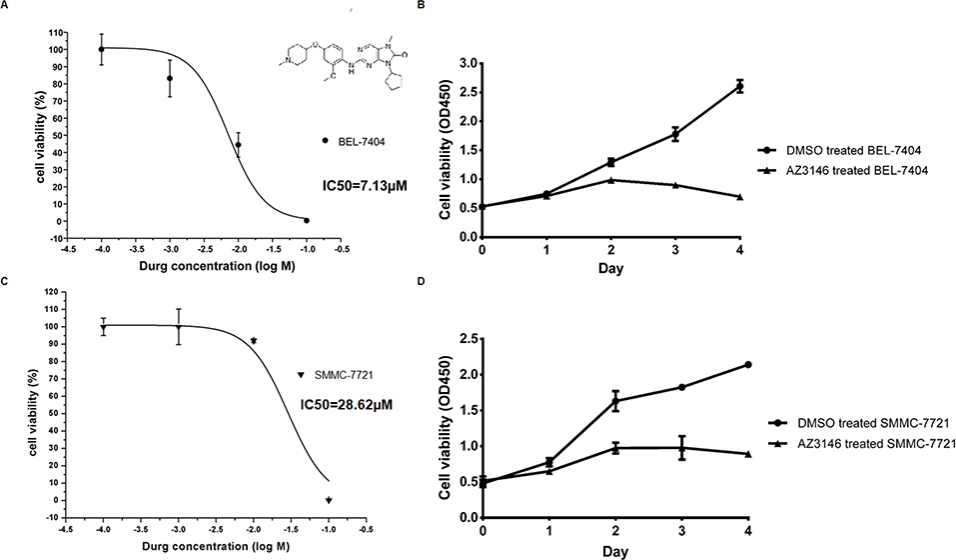
AZ3146 inhibits HCC cell growth *in vitro* **A.** and **C.** Dose response curve in BEL-7404 (A) and SMMC-7721 (C) with IC50 values, respectively. The molecular structure of AZ3146 was displayed in Figure 6A. **B.** and **D.** The growth curve of BEL-7404 (B) and SMMC-7721 (D) under the treatment of AZ3146 at the concentration of their IC50, respectively. Points, mean (*n* = 6); bars, SD.

Based on the results of clinicopathological analyses of TTK, we estimated its potential as a therapeutic target with the specific kinase inhibitor AZ3146. *In vitro* cell cytotoxicity assays were performed on SMMC-7721 and BEL-7404 cells. The 50% inhibiting concentration (IC50) values were calculated as being 7.13 μM (BEL-7404, Figure 6A) and 28.62 μM (SMMC-7721, Figure 6C). Both cells were further treated under the concentration of IC50 for 4 days. Significant inhibitions of cell proliferation were observed (Figure 6B and Figure 6D). The data suggest that TTK serves as a target for AZ3146 in HCC cells.

## DISCUSSION

In this study, we found TTK expression significantly correlated with histologic grade. It indicates that TTK contributed to HCC tumor progression. Screening on various normal tissues revealed that TTK was only expressed highly in the testis. It confirms the similar results identified by Mills et al. [19]. Meanwhile, we observed the strong expressions of TTK in human fetal livers and its absence in normal adult human livers. Taken together, we hypothesize TTK as a novel cancer-testis (CT)-related gene. It might contribute to carcinogenesis and tumor progression by hepatocytes de-differentiation to the embryonic state.

According to our data, other clinicopathological characteristics including tumor size, PVTT and distant metastasis were highly associated with TTK. It suggests that TTK stimulates HCC cellular growth and invasion. This hypothesis was convinced by results of *in vitro* gain and loss of function studies in HCC cells. Furthermore, TTK induced Akt/mTOR and MDM2/p53 signaling pathways with a wild-type p53 background. Considering the anchorage-independent growth ability of cancer cells, it indicates the role of TTK for increasing HCC cell aggressiveness [20]. In melanoma cells, TTK activates Akt and forms an auto-regulatory negative feedback loop with B-Raf^WT^/ERK signaling [8]. Besides Akt, TTK involves in several other signaling pathways. Activated TTK promotes TGF-β-independent Smad signaling activity through phosphorylating Smad2 and Smad3 [21]. Conversely, TTK functions at a point upstream of the MAPK and inhibits the Fus3 activation [22]. Whether TTK functions through these signaling pathways in HCC still needs more investigations.

Previous studies have revealed the correlation between CNV and the mRNA level of TTK in TNBC [11]. Despite of genetic alterations, aberrant methylation is another major mechanism for the dysregulation of oncogenes [23]. However, whether epigenetic alterations in cancer stimulate TTK expression is still unclear. Here we found the significant demethylation in TTK promoter of the HCC specimens. It demonstrats that epigenetic mechanism at least partly, induces TTK overexpression in liver cancer.

In our research, both TTK depletion and TTK activity inhibition significantly suppressed the viability of HCC cells. This finding strongly supports the data from Liang et al., in which TTK silencing restrains HCC cells resistance to Sorafinib [15]. The results exhibit the therapeutic potential of TTK in HCC treatment.

In conclusion, our data suggest the essential role of TTK in HCC carcinogenesis by promoting cell proliferation and migration, via activation of Akt/mTOR and MDM2/p53 signaling pathways. As a potential oncogene, TTK might serve as a prognostic marker and a therapeutic target valuable for HCC treatment.

## MATERIALS AND METHODS

### Tissue specimens and cell culture

All 152 HCC specimens and their adjacent normal liver specimens were obtained from patients underwent surgical operation at the Affiliated Hospital of Guilin Medical University in China. The diagnoses were described in our perivous study [24]. The pathological evaluations of the tissues were judged according to “Primary Liver Cancer Clinical Diagnosis and Staging Criteria” [25]. Six specimens of normal liver tissues surrounding the hepatic hemangioma tissues were collected. All of the normal tissues were verified by pathology after the operations. In addition, four cases of fetal tissues were taken from educed fetuses in the Department of Obstetrics in the Affiliated Hospital of Guilin Medical University in China. All of the samples above were frozen in liquid nitrogen and placed at −80°C immediately after the surgical resections. This study was approved by the ethics committee of Hospital Affiliated of Guilin Medical University. All patients provided their written informed consent to participate in this study according to the Declaration of Helsinki.

The following liver tumor-derived cell lines were used in this study: Huh7, SMMC-7721, PLC/PRF/5, YY-8103, QGY-7701, QGY-7703 and BEL-7404. All cell lines were cultured under Dulbecco's Modified Eagle's medium (DMEM; Sigma-Aldrich), supplemented with 10% FBS (Life Technology) in a 5% CO_2_-humidified chamber.

### Western blotting

Total cell proteins were extracted using cell lysate buffer (Cell signaling technology) with 1% Protease/Phosphatase Inhibitor Cocktail (Cell signaling technology). Equal amount of proteins were separated by 10% SDS-PAGE and transferred to nitrocellulose membranes (Amersham, GE Healthcare). Membranes with proteins were blocked by 5% non-fat milk in PBS buffer and then incubated with primary antibodies overnight at 4°C. The specific primary antibodies were as follows: Akt antibody (#C67E7, rabbit monoclonal antibody, dilution 1:1000, Cell Signaling technology), p-Akt (S473) antibody (#4060, rabbit monoclonal antibody, dilution 1:1000, Cell Signaling technology), mTOR antibody (#2972, rabbit polyclonal antibody, dilution 1:1000, Cell Signaling technology), p-mTOR (S2481) antibody (#2974, rabbit polyclonal antibody, dilution 1:1000, Cell Signaling technology), MDM2 antibody (#YT2690, rabbit polyclonal antibody, dilution 1:1000, ImmunoWay), p-MDM2 (S166) antibody (#3521, rabbit polyclonal antibody, dilution 1:1000, Cell Signaling technology), p53 antibody (#9282, rabbit polyclonal antibody, dilution 1:1000, Cell Signaling technology), p-p53 (S6) antibody (#9285, rabbit polyclonal antibody, dilution 1:1000, Cell Signaling technology), and Flag-tag antibody (#F3165, mouse monoclonal antibody, dilution 1:1000, Sigma-Aldrich). β-actin antibody (#4970, rabbit polyclonal antibody, dilution 1:1000, Cell Signaling technology) was used as the internal positive control. The trace of proteins were scanned after incubated with secondary antibodies conjugated with DyLight fluorescent dyes using Odyssey Infrared Imaging system (LI-COR) and quantitatively measured with Quantity One software.

### Semi-quantitative PCR

Each frozen tissue was cut into pieces of 20–30mg weight and ground to smash under the liquid nitrogen before RNA extraction. Total RNA was extracted using Trizol reagent (Takara), according to the recommended manual. The concentrations of total RNA were measured using NanoDrop 2000 (Thermo).

Reverse transcription was performed with 1 μg Total RNA using PrimeScript™ RT-PCR Kit (Takara), following the manufacturer's protocal to prevent the genomic DNA contamination.

Semi-quantitative PCR was performed using the following primers for TTK: 5′-TCCCCAGCGCAGCTTTCTGTAGA-3′ (forward) and 5′- CCAGTCCTCTGGGTTGTTTGCCAT-3′ (reverse). β-actin was measured as the internal reference with the following primers: 5′-AATCGTGCGTGACATTAAGGAG-3′ (forward) and 5′- ACTGTGTTGGCGTACAGGTCTT -3′(reverse). Lengths of the amplified sequences were 219bp (TTK) and 273bp (β-actin). The PCR reaction was performed in a volume of 20 μl using the TaKaRa PCR Kit. The reactions were performed at 94°C for 5 min followed by 34 cycles (for TTK) or 22 cycles (for β-actin) of 94°C for 30 sec, 55°C for 30 sec and 72°C for 30 sec. The PCR products were separated on 2% agarose gels containing ethidium bromide.

### mRNA quantitation

The mRNA expression of TTK in HCC specimens and their adjacent non-tumourous liver tissues were quantified using QuantiGene Plex 2.0^®^ assay (Affymetrix) following the manufacturer's instructions. All the oligo nucleotide probe sets including capture, label and blocker probes were designed by the manufacturer. For each sample, 200 μg extracted total RNA was used at the beginning of the assay. Mean fluorescence intensity (MFI) generated from each specific probe set were captured and quantified using a Luminex instrument (Bio-Rad).

### Ectopic expression of TTK and RNAi

The full length of TTK CDS (2574bp, NM_003318) was amplified by PCR from the cDNA of the liver tumor derived cell line SMMC-7721. The primer sequences were designed as follows: 5′-TAGGATCCGCCACCATGGAATCCGAGG ATTTAAGT-3′ (forward) and 5′-GGCTCGAGTTTTTTTCCCCTTTTTTTTTC-3′ (reverse). The PCR products were cloned into the flag-tagged pCMV-3Tag-3 vectors (Agilent Technologies). The reconbinant TTK-pCMV-Flag plasmids were transformed into the competent cell DH5α, and extracted using NucleoSpin Plasmid extraction kit (MACHEREY-NAGEL).

The siRNA against TTK and the negative control (NC) were designed and chemically synthesized (Shanghai GenePharma) as follows: TTK: 5′-GUGGCAGAGAAUUGACAAUTT-3′ (sense) and 5′-AUUGUCAAUUCUCUGCCACTT-3′ (antisense); NC: 5′-UUCUCCGAACGUGUCACGUTT-3′ (sense) and 5′-ACGUGACACGUUCGGAGAATT-3′ (antisense). The siRNAs worked at the concentration of 100nM. Both plasmids and siRNAs were transfected into cells using Lipofectamine 2000 transfection reagent (Invitrogen) according to the manufacturer's instruction.

### Cell proliferation assay

The “xCELLigence” system (ACEA Biosciences) and the Cell Counting Kit-8 (CCK-8; Dojindo Laboratories) were both applied to monitor cell growth responses.

In The “xCELLigence” system, a label-free measurement of cells is performed in the RTCA-DP instrument. The cellular status is displayed as the dimensionless parameter “Cell Index” (CI) [26]. After transfection with plasmids or siRNAs for 24 h, HCC cells were stripped and replated into the E-plates at the density of 7.5 × 10^3^ per well. E-plates were then transferred to the RTCA instrument for automated real-time monitoring at standard incubator conditions, with quadruplet read-outs of CI every 60 min the following 6 days. The experiments were independently repeated 2 times.

For cell viability assessment using CCK-8, 3 × 10^3^ cells per well were seeded into 96-well plates. CCK-8 was applied following the manufacturer's protocal at a 24-hour interval for 3–7 days. The absorbance was measured at 450nm as an indicator of cell viability. The experiments were independently repeated 3 times.

### Soft agar colony formation assay

TTK-pCMV-Flag plasmids were transfected into HCC cell lines (empty vector as a control). After 24 hours, the transfected cells were stripped and replated into 24-well plates containing 1% base agar and 0.5% top agar, at the density of 2 × 10^3^ cells per well. Plated cells were incubated at 37°C for 21 days with a 100 μl culture medium addition every 4–5 days. Colonies were counted and photographed under a microscope (magnification, × 40). The experiments were independently repeated 3 times.

### Cell migration assay

Cell migration assay was performed using 24-well Transwells (8 μm pore size; BD Biosciences). Each well of the lower chamber was filled with 500 μl DMEM containing 10% FBS. HCC cells were starved in serum-free medium overnight. A total of 1 × 10^5^ HCC cells in 500 μl DMEM containing 1% FBS were plated into each well of the upper chamber. After an incubation for 48 h, the cells were fixed with 4% paraformaldehyde for 15 min and stainned with 0.5% crystal violet for at least 30 min. Cells remaining in the upper chamber were removed by cotton swabs. Cells on the lower surface of the membrane were counted and photographed for at least 3 random microscopic fields (magnification, × 40). The experiments were independently repeated 3 times.

### Bisulfite sequencing

Genomic DNA was extracted from the paired HCC tumor and the adjacent non-tumor specimens using the Dneasy Tissue kit (Qiagen) before being treated with the EpiTect Bisulfite Kit (Qiagen) according to the manufacturer's instructions. The CpG island of TTK was estimated at the EMBL-EBI Web Server (http://www.ebi.ac.uk/Tools/seqstats/) and amplified with the following primers: 5′-GTTTAAGGTGTATTTTTTAGG-3′ (forward) and 5′-AAAAATAAAACCCAAACTCCTTTC-3′ (reverse). The products of the PCR were purified and cloned into the pMD18-T vector (TaKaRa). 5–10 colonies of each sample were randomly picked for sequencing.

### Inhibitor treatment to HCC cells

The TTK inhibitor AZ3146 (Selleck) was disolved in DMSO at a concentration in 100 mM and diluted into 100 μM, 10 μM, 1 μM and 0.1 μM sequentially with DMEM containing 10% FBS before use. *In vitro* cytotoxicity assays were performed as described previously [27]. HCC cells were plated into 96-well plates at the density of 3 × 10^3^ per well. AZ3146 was added in the indicated concentrations the next day. The inhibitor treated cells were cultured and tested at a 24-hour intervals for 3–4 days using CCK-8.

### Statistical analysis

The statistical analyses were performed using SPSS21.0 software. The cut-off value (0.226) was optimized using Medcalc software for defining high and low subgroups following this paper [28]. The correlation analysis was performed using the Chi-squre Test. The survival analysis was performed using the Kaplan-Meier method with the Log-Rank Test for statistical significance check. The two-tailed Student's *t*-test was used to evaluate the significance of quantitative variables. The IC50 values were calculated with Origin 8.0 software. *P* ≤ 0.05 was defined as statistical significance.

## SUPPLEMENTARY TABLE


