# Supplementary material for: TTK activates Akt and promotes proliferation and migration of hepatocellular carcinoma cells
Source: Oncotarget. 2015 Sep 19;6(33):34309–20. doi: 10.18632/oncotarget.5295 (PMC4741454; doi:10.18632/oncotarget.5295)
Supplement: Supplementary file 1 [file oncotarget-06-34309-s001.pdf]

SUPPLEMENTARY TABLE

Supplementary Table S1: Peripheral blood AFP and TTK expression in HCC patients under age 50

SUM	Patients' categories			
	TTK high[a]/AFP high[b]	TTK low/AFP high	TTK high/AFP low	TTK low/AFP low
No.of patients *n* (%)	29 (38.67)	17 (22.67)	18 (24.00)	11 (14.67)
Diagnostic rate 1 (%)	61.33		--	
Diagnostic rate 2 (%)	85.33			---------------------

[a]high of TTK mRNA expression in HCC specimens was designed as greater than or equal to 0.226

[b]high of peripheral blood AFP was designed as greater than or equal to 200 μg/l
